# COVID-19 related mortality in older adults: analysis of the first wave in Colombia and Mexico

**DOI:** 10.26633/RPSP.2021.109

**Published:** 2021-09-01

**Authors:** Mario Ulises Pérez-Zepeda, Santiago Campos-Fajardo, Carlos Cano-Gutierrez

**Affiliations:** 1 Instituto Nacional de Geriatría Mexico City Mexico Instituto Nacional de Geriatría, Mexico City, Mexico; 2 Pontificia Universidad Javeriana Bogotá Colombia Pontificia Universidad Javeriana, Bogotá, Colombia; 3 Hospital Universitario San Ignacio Bogotá Colombia Hospital Universitario San Ignacio, Bogotá, Colombia

**Keywords:** COVID-19, epidemiology, aged, cross-cultural comparison, Colombia, Mexico, COVID-19, epidemiología, anciano, comparación transcultural, Colombia, México, COVID-19, epidemiologia, idoso, comparação transcultural, Colômbia, México

## Abstract

**Objective.:**

The aim of this study is to describe the mortality among older adults in the first wave of COVID-19 in Colombia and Mexico.

**Methods.:**

This is an observational, prospective study on data obtained from open data sets that are publicly available on the websites of the health ministries of the respective countries. COVID-19 cases, age, sex, date to mortality, and mortality itself were analyzed with Kaplan-Meier curves and Cox regressions.

**Results.:**

Data on 1 779 877 individuals were analyzed, 58.2% from Mexico, with a higher frequency of men for both countries, and 11.7% were older adults. Survival curves show a continuous increase in mortality for Mexico, with higher rates for older adults, while for Colombia the mortality was observed up to 50 days of the follow-up. Finally, hazard ratios were higher for older adults in both countries. Colombia implemented a rigid curfew for older adults, and the effect on mortality is clear from the survival curves.

**Conclusions.:**

This finding shows the potential benefit that public policies could have on older adults.

## RESUMEN

**Objetivo.:**

El propósito de este estudio es describir la mortalidad en personas mayores durante la primera ola de la COVID-19 en Colombia y México.

**Métodos.:**

Se trata de un estudio prospectivo observacional realizado sobre la base de la información obtenida de los conjuntos de datos abiertos disponibles públicamente en los sitios web de los ministerios de salud de estos países. Se analizaron los casos de COVID-19, la edad, el sexo, la fecha de mortalidad y la propia mortalidad con curvas de Kaplan-Meier y regresiones de Cox.

**Resultados.:**

Se analizaron los datos de 1 779 877 personas, 58,2% de México, con una mayor frecuencia de hombres en ambos países y 11,7% de los cuales eran personas mayores. Las curvas de supervivencia muestran un incremento continuo de la mortalidad en México, con las tasas más altas en personas mayores, mientras que en el caso de Colombia se observó la mortalidad hasta 50 días durante el seguimiento. Por último, las razones de riesgo fueron mayores en las personas mayores de ambos países. Colombia puso en marcha un estricto toque de queda para las personas mayores y su efecto sobre la mortalidad resulta patente en las curvas de supervivencia.

**Conclusiones.:**

Estos resultados muestran los posibles beneficios de las políticas públicas dirigidas a las personas mayores.

## RESUMO

**Objetivo.:**

O objetivo deste estudo é descrever a mortalidade entre idosos na primeira onda de COVID-19 na Colômbia e no México.

**Métodos.:**

Este é um estudo prospectivo observacional com dados obtidos de conjuntos de dados abertos disponíveis publicamente nos sites dos ministérios da saúde dos respectivos países. Foram analisados casos de COVID-19, idade, sexo, tempo até a morte e mortalidade em si, usando curvas de Kaplan-Meier e regressões de Cox.

**Resultados.:**

Foram analisados dados de 1 779 877 indivíduos, 58,2% deles do México, com uma maior frequência de homens nos dois países; 11,7% eram idosos. As curvas de sobrevida mostram um aumento contínuo da mortalidade no México, com taxas mais altas para idosos, ao passo que na Colômbia a mortalidade foi observada dentro de um período de até 50 dias de acompanhamento. Além disso, nos dois países as razões de risco foram mais altas para idosos. A Colômbia implementou um toque de recolher rigoroso para os idosos, e o efeito sobre a mortalidade é evidente com base nas curvas de sobrevida.

**Conclusões.:**

Este achado demonstra o potencial benefício que as políticas públicas poderiam ter para os idosos.

The coronavirus disease 2019 (COVID-19) pandemic has highlighted ancient problems in the health systems of the world, one example of which has been the death toll in older adults. In addition, it has brought into question several paradigms that were thought to be solid but are still problematic in our times. Moreover, it has also evidenced the long-standing problem of ageism—prejudice based solely on the number of years since the day a person was born—which is the most widely used parameter to deny treatments when facing scarcity (1). Since the beginning of the pandemic, older adults were found to be at higher risk of not only being infected but also dying from the disease (2). Evidence continues to accumulate regarding the epidemiological characteristics of COVID-19, corroborating that case-fatality rate increases with age (3). However, it is not clear whether age is an accurate predictor of adverse outcomes in individuals with COVID-19, and what role other factors play at the country level: age-specific policies, restrictions, better health services for this age group, social networks, etc.

When comparing the mortality ratio globally, Latin American countries have among the world’s highest rates. A share of inequality and corruption might explain the heavy burden that this pandemic is implying for countries of the region. For example, Mexico faced its first wave, with a short pause, now fully merged into its second wave. In contrast, Colombia had lower consequences in its first wave but now is also facing an increase both in cases and deaths. Evidence from the region and around the world on the impact of age on mortality is needed and will contribute to a rethink of policies targeting older adults, not only at the present, but also those policies that could impact in the long term and even in preparing for new pandemics (4). The objective of this work is to describe COVID-19-related mortality in the first wave of the pandemic in two countries of the Latin America region, Colombia and Mexico, with a focus on the impact of age and the differences between the approaches of each country in tackling the pandemic.

## MATERIALS AND METHODS

### Setting and design

This study is a secondary analysis of the COVID-19 surveillance system data of the Colombian and Mexican ministries of health. These established systems have been gathering data from the current pandemic, with similar variables available (5) (https://www.gob.mx/salud/documentos/datos-abiertos-152127). Individuals with suspected COVID-19 infection, based on symptoms, are included in this surveillance system. For the purposes of this analysis, the first cases included are from the month of February and the last ones from 30 September, amounting to 216 follow-up days. Having this range of data accounts for the most part of the first wave of the outbreak in both countries.

### Sample

Individuals could be included from all health systems and private practices, in both countries. Detailed description on this is available in the websites of both ministries of health, from Colombia (https://covid19.minsalud.gov.co/) and Mexico (https://coronavirus.gob.mx/). In brief, testing for COVID-19 was done using nasal swabs performed at specialized sites. As previously stated, regardless of the test result, these individuals were followed from first contact through every stage of their disease until death or recovery.

### Variables

In order to achieve our objective, variables used in this work included: age, sex, follow-up days, status (alive or deceased), and country (Colombia or Mexico). Other variables available in the data sets but not included in our analyses were: place of residence, speaking an indigenous language, migration status, nationality, site of reference, and pregnancy. Age was categorized into older adult (≥65 years) or not.

### Statistical analysis

Descriptive statistics were done using relative and absolute frequencies for those categories examined. Survival analyses were performed, including individuals who survived up to the final assessment or died. Kaplan-Meier curves were constructed, and the log-rank test assessed the difference between variables. Multivariate Cox regression models examined the association of age with mortality, stratified by country and adjusted for sex. Finally, log-log graphs were assessed in order to test the proportional risk assumptions.

### Ethical issues

Amid the COVID-19 pandemic, the need for new evidence surpasses the availability of ethics committees, particularly in the case of non-interventional protocols and observational studies, where the risk for individuals is minimal. The Mexican epidemiological surveillance system is governed by the Mexican *Open Data* law, and all individuals are protected by this law. A similar approach applies to the Colombian data. Data sets were de-identified and informed consents were obtained.

## RESULTS

From a total of 1 779 877 individuals, 41.8% were from Colombia (*n* = 744 137) and 58.2% from Mexico (*n* = 1 035 740). The mean age of the Colombian sample was 39.7 years (± standard deviation [SD] 18.1) and for Mexico it was 44 years (± SD 16.8). There was a higher frequency of men in both countries (50.6% average). Mortality for the general population was higher in Mexico at 9.1% (*n* = 94 853) than in Colombia at 3.1% (*n* = 22 807). The average frequency of mortality for older adults in both countries was 29.2% (*n* = 60 771), with a significant difference between countries, being higher in Mexico at 35.1% (*n* = 45 901) than in Colombia at 19.2% (*n* = 14 870). Specifically, there were higher rates of death in men and older adults in both countries (Table 1).

**FIGURE 1.**
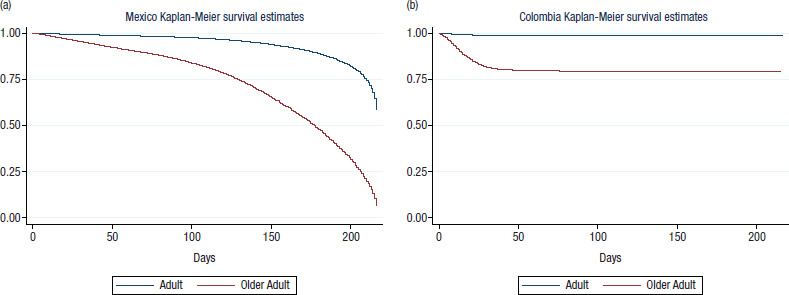
Kaplan-Meier survival estimates, a) Mexico b) Colombia

***Note:*** Log-rank test *p*-value < 0.001

***Source:*** Prepared by the authors based on their analysis of the study data.

As shown in Figure 1, the time-to-event rate of mortality was higher in older adults from both countries, with a continuous trend for Mexican individuals, and an initial high number of deaths in Colombia with stagnation of the curves later. Both Kaplan-Meier curves were different with a statistical significance of *p* < 0.001.

Finally, in the Cox regression models, older adults have a higher strength of association with mortality, even when adjusting for sex (Table 2). For Colombia, the hazard ratio was 18.18 (95% confidence interval [CI] 17.68, 18.69; *p* < 0.001) and for Mexico 6.26 (95% CI 6.18, 6.34). Men had higher hazard ratio in both countries.

## DISCUSSION

Colombia and Mexico currently are two countries that share a high toll of COVID-19 cases. Moreover, at the time of writing, it is to be expected that in the coming days and weeks, health services will be flooded by COVID-19 cases, and triage will be put in place. It is our hope that an appropriate assessment independent of age will be implemented. In fact, as we write this article, the second wave is evidencing higher death rates in both countries, despite policies being implemented to diminish the impact of the pandemic. Increasing demand for hospital care is implicated among the causes of higher mortality, as capacity in both countries is limited, which could affect the care of those seeking help in these sites. Along with this, it is now well known that age is one of the main risks for high mortality; however, our findings suggest that this mortality can be diminished when appropriate public policies are put in place targeting older adults.

To the best of our knowledge, this is the first work to report on mortality in two Latin American countries with a focus on older adults. Surveillance data show that Latin America is experiencing a high toll imposed by this catastrophe (6). Moreover, this crisis is particularly impacting older adults, reflecting the need for stronger actions to protect this vulnerable population group.

There are several factors, including inequality, that could explain the higher rates of mortality in older adults (7–11), such as clinical judgement to triage individuals with a better possibility of surviving; however, so-called “classical” tools to stratify patients have shown to have low predictive ability in those suffering from COVID-19 (12). On the other hand, the probable defining factor for mortality is the morbidity burden rather than age. However, it is unclear if individual conditions (e.g., kidney failure, obesity, diabetes) have different impacts in younger adults than older adults (13). This is particularly important for Mexico as it has a high prevalence of diabetes, but Colombia also has a high frequency of metabolic problems in its population. It is noteworthy that older adults have additional problems, such as frailty, which are frequently overlooked or erroneously mistaken for multimorbidity.

**TABLE 1.** General description of the sample, stratified by mortality

	Both countries (*N* = 1 779 877)			Colombia (*n* = 744 137)			Mexico (*n* = 1 035 740)		
	Total	Deceased (*n* = 117 660)	Alive (*n* = 1 662 217)	Total	Deceased (*n* = 22 807)	Alive (*n* = 721 330)	Total	Deceased (*n* = 94 853)	Alive *(n* = 940 887)
Age (Mean (SD))	42.2 (17.5)	64.2 (14.5)	40.6 (16.6)	39.7 (18.1)	68.8 (15.1)	38.8 (17.5)	44 (16.8)	63 (14.1)	42 (15.8)
Older adult (*n* (%))	208 188 (11.7)	60 771 (29.2)	147 417 (70.8)	77 276 (10.4)	14 870 (19.2)	62 406 (80.8)	130 912 (12.6)	45 901 (35.1)	85 011 (64.9)
Female (*n* (%))	879 855 (49.4)	43 102 (4.9)	836 753 (95.1)	367 430 (49.4)	8 205 (2.2)	359 225 (97.8)	512 425 (49.5)	34 897 (6.8)	477 528 (93.2)

***Source:*** Prepared by the authors based on their analysis of the study data.

**TABLE 2.** Cox regression stratified by country, including age and sex as independent variables

	Colombia		Mexico	
	Hazard ratio (95% CI)	*p*-value	Hazard ratio (95% CI)	*p*-value
Older adult	18.18 (17.68, 18.69)	<0.001	6.26 (6.18, 6.34)	<0.001
Male	1.74 (1.69, 1.79)	<0.001	1.46 (1.40, 1.48)	<0.001

***Source:*** Prepared by the authors based on their analysis of the study data.

In catastrophic situations like the COVID-19 pandemic, public health ethics prevails over medical ethics (a more person-centered approach) (14), with the general well-being of the population as the goal (15). This usually includes allocating scarce resources to the most cost-effective interventions (16, 17). Even though this decision-making process is always based on the available evidence, it is in times like this that decisions must be made based on past experience and not on assumptions or prejudices (18, 19). It is our understanding that age-based decisions are founded in the idea that age is a unique factor for mortality, especially in critical care. Certainly, age is an important risk factor, but it is not the only one; multimorbidity is just as important, if not more so (20). Often, this bias toward older age comes from a preconceived notion of what older age means and what it looks like, and that a younger, more productive population is necessary.

Since most individuals affected by COVID-19 are older adults, a better characterization of the population will result in better strategies for their care. For example, frailty should be a mandatory characteristic gathered in these epidemiological studies (and daily clinical routine), as it has a highly predictive value for mortality in several settings, including critical care (21–23). This would certainly shift the focus from the number of years of age and would allow for a more objective and fair allocation of resources (24). Of course, this is not enough, but it is certainly a good start.

It is well known that underweight older adults have higher rates of mortality than obese ones (25), although it is not clear how the variable “obesity” was measured in those data. The optimal method for evaluating this association in older adults is to use measurements of body composition that consider muscle mass—a better predictor of adverse outcomes in older adults (26). Adding parameters of muscle mass and muscle function will certainly consider the “normality” of aging.

An important finding of our study can be seen in the survival curves, where it is evident how the death rate in older adults was halted in Colombia by the 50th day approximately; the rate in the younger population was steadily low for Colombia, and this might explain the higher hazard ratio for these individuals. Moreover, the abrupt stop in mortality for this country most likely is explained by several policies that were implemented early in the pandemic. These measures have been based on government resolutions, circulars, and decrees from 10 March to date, November 2020. The government decided on the “preventive isolation to protect adults over 70 years of age” through Resolution 464 on 18 March 2020, since this group was considered in that country to be at highest risk (27). Likewise, it adopted mandatory health measures for the preventive isolation of older adults in long-stay centers and for the partial closure of activities in life centers and day centers on 20 March 2020 (28). On 24 March 2020, the mandatory preventive isolation began by Decree 457 34, which ended on 31 August. As of 1 September 2020, Colombia ended the government-imposed quarantine and began a new phase of isolation, called selective isolation. These measures were enforced by the armed forces and were effective, as shown in the mortality curve. On the other hand, in Mexico there was never a policy in place specifically to protect older adults, and policies on this matter have been rather ambiguous and not enforced by the authorities.

### Limitations

This study has a few limitations. We used data that are constantly changing, so our data may represent only a trend and should be explored at different stages. In addition, data may fall into an ecological fallacy if trying to compare both countries; instead, our intention was only to describe the situation in similar countries of the same region. In addition, our models and estimates are limited to the variables examined, excluding some others, which could make the interpretation of our results difficult. For example, there was no information available on the prognostic importance of imaging in COVID-19 (29).

### Conclusion

Older age is an important factor when it comes to mortality and, as shown by our results, there was a significant difference between younger and older adults in both countries. It is important to stress the importance of having in place selective policies for those who are at higher risk, and our findings support the fact that, even between similar countries, attitudes adopted by governments can influence the outcomes in a health crisis, such as the COVID-19 pandemic.

## Disclaimer.

Authors hold sole responsibility for the views expressed in the manuscript, which may not necessarily reflect the opinion or policy of the *RPSP/PAJPH* and/or PAHO.
